# Influence of Sex on Therapeutic Adherence in Cardiovascular Diseases: A Scoping Review

**DOI:** 10.3390/jcm14124253

**Published:** 2025-06-15

**Authors:** Guillermo Moreno, Blanca Moreno-Ferreiro, Carla Pérez-Ingidua, María Jesús Vicente-Galán, Verónica Gimeno-Hernán, Elena Orgaz-Rivas, María José González-Sanavia, Ana Belén Rivas-Paterna, Enrique Pacheco del Cerro, Alfonso Meneses-Monroy

**Affiliations:** 1 Departamento de Enfermería, Facultad de Enfermería, Fisioterapia y Podología, Universidad Complutense de Madrid (UCM), 28040 Madrid, Spain; blamor02@ucm.es (B.M.-F.); carlap05@ucm.es (C.P.-I.); mvicen14@ucm.es (M.J.V.-G.); verogime@ucm.es (V.G.-H.); elenaorg@ucm.es (E.O.-R.); marjgonz@ucm.es (M.J.G.-S.); ab.rivas@enf.ucm.es (A.B.R.-P.); quique@ucm.es (E.P.d.C.); ameneses@ucm.es (A.M.-M.); 2 Grupo de Investigación Cardiovascular Multidisciplinar Traslacional (GICMT), Área de Investigación Cardiovascular, Instituto de Investigación Hospital 12 de Octubre (imas12), 28041 Madrid, Spain; 3 Servicio de Farmacología, Hospital Universitario Clínico San Carlos, 28040 Madrid, Spain; 4 Consulta de Insuficiencia Cardiaca, Hospital de Día, Servicio de Medicina Interna, Hospital Universitario Fundación Alcorcón, 28922 Madrid, Spain; 5 Processes, Research, Innovation and Information Systems Unit, Directorate of Nursing, Instituto de Investigación Sanitaria San Carlos (IDISSC), Hospital Clínico San Carlos, 28040 Madrid, Spain

**Keywords:** sex, therapeutic adherence, cardiovascular disease, risk factors, sex inequalities

## Abstract

**Background/Objectives:** Females with cardiovascular disease (CVD) are often misdiagnosed, and they have sex-related psychosocial risk factors that pose specific health risks and affect their adherence to treatment. This study aims to evaluate sex differences in adherence to, and risk prediction for, secondary prevention measures in patients with cardiovascular disease. **Methods:** A scoping review of the literature was conducted. A search strategy was carried out in the PubMed, Scopus, and Web of Science databases. Articles were selected according to PRISMA guidelines, focusing on studies published within the last five years involving patients with cardiovascular disease and written in English or Spanish. Bias was assessed using the CASPe questionnaire. This project has been registered in the Open Science Framework (OSF) repository under the DOI code 10.17605/OSF.IO/GYDZF. **Results:** Thirteen articles were retrieved. For hypertension, medication adherence ranges from 25% to 83% in males and from 24% to 80% in females. For ischemic heart disease, the range is 32–74% in males and 32–60% in females. Adherence to physical activity ranges from 21% to 72% in males and 14% to 72% in females. Predictive factors include older age, increased comorbidity, and psychosocial aspects. **Conclusions:** There is evidence of sex differences in medication adherence for hypertension, ischemic heart disease, and peripheral arterial disease. However, further research is required to identify the factors that predispose individuals to non-adherence.

## 1. Introduction

Cardiovascular diseases (CVDs) remain the leading cause of death worldwide, accounting for around 17.9 million deaths each year [1]. In Spain alone, CVDs were responsible for 26.5% of all deaths in 2023 [2], illustrating the ongoing public health challenge posed by these diseases. There is increasing attention being given to sex-specific differences in both incidence and outcomes, revealing that females often experience delayed diagnoses, receive suboptimal treatment, and display distinct patterns of therapeutic adherence compared to males [3]. While males are more frequently diagnosed with ischemic heart disease, females are more commonly affected by cerebrovascular diseases and heart failure with preserved ejection fraction [4].

Cardiovascular risk factors (CVRFs), including hypertension, dyslipidemia, type 2 diabetes mellitus (T2DM), obesity, smoking, and physical inactivity, vary in prevalence and impact by sex [5,6]. For instance, hypertension is more prevalent among postmenopausal females and is associated with increased arterial stiffness and left ventricular hypertrophy [7]. Obesity confers a higher relative risk of CVD in females than in males, potentially due to differences in fat distribution and hormonal influences [8]. Although T2DM is more prevalent in males, it significantly increases the risk of CVD and mortality in females [9,10]. Dyslipidemia patterns also differ; males more often exhibit elevated LDL cholesterol, while postmenopausal females show reduced HDL cholesterol levels [11].

Behavioral factors such as smoking and physical inactivity are also influenced by sex. Females are often more sedentary and more sensitive to the cardiovascular effects of tobacco, particularly when combined with the use of hormonal contraceptives [12]. Moreover, societal roles and caregiving responsibilities may restrict female’s ability to engage in health-promoting behaviors such as regular exercise or dietary regulation [13,14].

In addition to the traditional CVRFs, females are exposed to sex-specific factors that contribute to CVD risk. These include hormonal fluctuations during the menstrual cycle, complications related to pregnancy such as gestational diabetes and pre-eclampsia, and menopause [11]. Complications during pregnancy have been linked to long-term cardiovascular risk [15]. For instance, pre-eclampsia has been linked to an increased risk of hypertension and ischemic heart disease later in life [16]. Similarly, early menopause correlates with a higher risk of CVD, likely due to the loss of the vasoprotective effects of estrogen [17].

Hormone replacement therapy (HRT) has been studied as a means of mitigating postmenopausal CVD risk. Evidence suggests that HRT may offer cardiovascular protection when initiated early in menopause; however, the benefits and risks remain controversial and must be considered on a case-by-case basis [18].

Sex-based social inequalities further increase the risk of CVD. Females face structural barriers such as underrepresentation in clinical trials, sex bias in diagnostic criteria, and disparities in access to healthcare [19,20]. The ‘Yentl syndrome’ describes the phenomenon whereby females receive equitable treatment only when presenting with symptoms typically experienced by males [21]. Furthermore, economic dependency and caregiving burdens often cause females to prioritize family responsibilities over their own health, which affects their ability to adhere to medical advice [22].

Therapeutic adherence is defined as the extent to which patients follow agreed medical regimens. Non-adherence has been linked to increased hospitalizations, disease progression, and healthcare costs [23]. The World Health Organization categorizes adherence as a multidimensional phenomenon influenced by factors relating to the patient, their condition, their therapy, the health system, and the socioeconomic context [24].

In the context of CVD, adherence to pharmacologic therapy (e.g., antihypertensives, statins, and antiplatelet agents) and lifestyle modifications (e.g., diet, physical activity, and smoking cessation) is essential. Numerous studies show that females are less likely than males to adhere consistently, particularly with regard to medication management and participation in cardiac rehabilitation [25]. Although recent meta-analyses on hypertension have indicated that sex is not significantly associated with differences in adherence to antihypertensive medications [26,27], other studies have reported higher rates of non-adherence among males [28]. Additionally, some reviews have demonstrated that sex plays a role in adherence to lipid-lowering therapy in patients with atherosclerotic cardiovascular disease [29], as well as in both primary and secondary prevention settings [30] Furthermore, meta-analyses have shown that adherence to cardiac rehabilitation programs is significantly lower among females compared to males [31,32]. Limited evidence also suggests that females may exhibit poorer adherence following acute myocardial infarction and stroke. In the context of heart failure, however, adherence studies have yielded inconsistent results [25].

In order to design effective interventions, it is essential to understand the factors contributing to these sex disparities. Therefore, it is essential to review and analyze the factors contributing to this discrepancy in adherence. The aim of this study is to explore how the literature has addressed sex differences in adherence to and risk prediction related to secondary prevention measures in patients with cardiovascular disease.

## 2. Materials and Methods

### 2.1. Design

This scoping review explores quantitative and qualitative studies investigating the influence of sex on therapeutic adherence in cardiovascular disease. The focus was to map the existing evidence and identify the biological and sociocultural determinants of adherence in males and females, as well as the prevalence of adherence to secondary prevention measures in males and females.

### 2.2. Data Sources and Search Strategy

The research was conducted using the databases PubMed, Scopus and Web of Science (WOS). The search terms included Medical Subject Headings (MeSH) and free-text keywords: ‘sex differences’, ‘therapeutic adherence’, ‘patient compliance’, ‘cardiovascular disease’, ‘sex disparities’, and ‘treatment outcomes’. Boolean operators were used to effectively combine the terms. The inclusion period was limited to 2019–2024 to reflect the most current research. Filters were applied to include only peer-reviewed, full-text articles written in English or Spanish. The reference lists of the selected articles were manually searched to identify any additional eligible studies. The search strategy and the results obtained are presented in Table 1.

### 2.3. Inclusion and Exclusion Criteria

Studies were included if they focused on therapeutic adherence in adults diagnosed with any cardiovascular disease; provided sex-disaggregated results or analyses; employed quantitative or qualitative designs; and were published in English or Spanish between 2019 and 2024. Studies were excluded if they were not original research; did not focus on adherence or sex differences; or involved non-cardiovascular conditions.

### 2.4. Selection Process

The selection process adhered to the PRISMA guidelines [33]. The quality of each study was evaluated using the CASPe tool [34]. Only studies rated as high quality (score > 7/10) were included. All data were manually collected by two independent researchers (GM and BMF). The project has been registered in the Open Science Framework (OSF) repository under the DOI code 10.17605/OSF.IO/GYDZF.

## 3. Results

### 3.1. Overview of Included Studies

**Figure 1:**
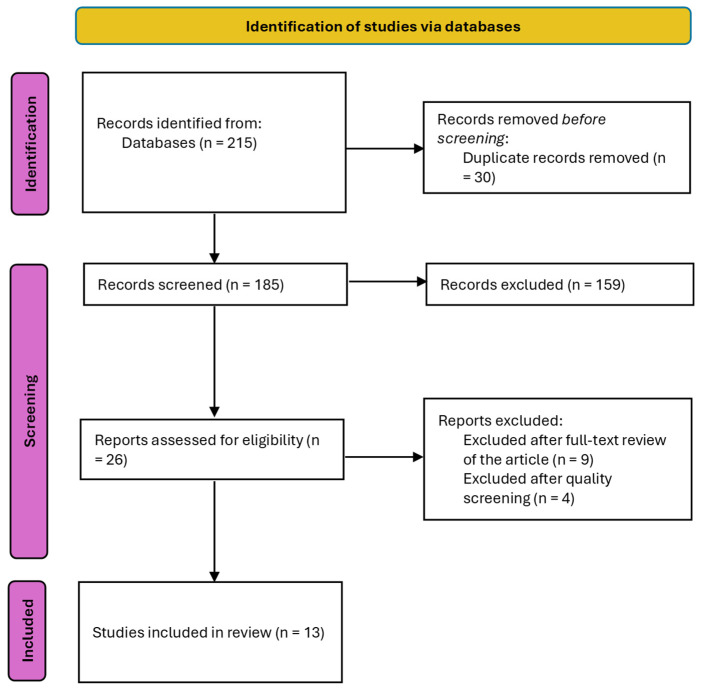
PRISMA flowchart: search results and study selection and inclusion process.

An initial pool of 215 articles was identified, of which 30 were duplicates and were therefore removed. Following title and abstract screening, 26 full-text articles were reviewed, of which 13 met all criteria (see Figure 1). The quality assessment of the included articles can be found in Supplementary Table S1.

The final sample comprised 13 studies, encompassing data from 10 countries and 1,103,905 individuals (see Table 1). The mean female representation was 28.4%, reflecting the ongoing underrepresentation of females in cardiovascular research. The studies addressed three categories CVD: ischemic heart disease (n = 8), hypertension (n = 4), and peripheral arterial disease (n = 1). The results on adherence and associated predictors for each type of secondary prevention, disaggregated by sex, are presented in Table 2.

### 3.2. Adherence to Pharmacological Treatment

Regarding hypertension, males exhibited slightly higher adherence rates, ranging from 25% to 83%, compared to 24% to 80% for females. Adherence was influenced by age: younger females demonstrated the lowest adherence, while elderly females often showed improved compliance, potentially due to increased awareness and healthcare utilization. For ischemic heart disease, medication adherence rates ranged from 32% to 74% for males and from 32% to 60% for females. Females were less likely to receive dual antiplatelet therapy, beta-blockers, and statins (71% vs. 78%, respectively). Over time, females with ischemic heart disease have received less secondary prevention care and have shown worse outcomes compared to males (Hyun et al. [39]). In relation to peripheral artery disease, one study reported adherence rates of 71% in males and 59% in females.

### 3.3. Adherence to Lifestyle Modifications

Adherence to physical activity guidelines was consistently lower among females, ranging from 14% to 72%, compared to 21% to 72% among males. Only three studies reported adherence to the Mediterranean diet, with highly variable values: among males, adherence ranged from 13% to 56%, and among females from 9% to 55%. Enrolment and completion rates for cardiac rehabilitation programs were significantly lower in females. For males, the range was 29–45%, and for females, it was 20–35%. However, females demonstrated slightly better smoking cessation rates. The analyzed studies report adherence rates of around 43% for males, while adherence among females increases to approximately 50%, regardless of the specific condition examined. Adherence to scheduled follow-up appointments was markedly lower among females, with some studies reporting rates below 30%.

### 3.4. Predictors of Poor Adherence

Among patients with ischemic heart disease, non-adherence to pharmacological treatments is more common among those followed by cardiologists than by primary care physicians. Key clinical predictors include a history of acute myocardial infarction, elevated LDL-C levels, diabetes, chronic kidney disease (CKD), hypertension, and an overall higher disease burden. Females, especially younger ones, showed significantly lower adherence rates. In the case of hypertension, the key predictors of non-adherence were identified as younger age, low disease awareness, low self-efficacy, unemployment, low income, absence of comorbidities, and female sex. Psychological comorbidities, such as depression, were also more prevalent among females, further contributing to lower adherence. In peripheral arterial disease, female sex, the use of moderate- or low-intensity statin therapy, and the presence of anxiety and depression were associated with lower adherence, whereas older age was a protective factor.

In patients with ischemic heart disease, non-adherence to a healthy diet was associated with CKD and poor control of cardiovascular risk factors, as well as obesity, being overweight, and having a higher number of comorbidities. Female sex was again linked to lower adherence. However, adherence improved with a previous Mediterranean lifestyle, a better quality of life, and strong family support. Among hypertensive patients, poorer dietary adherence was associated with rural residence and low self-efficacy.

In patients with ischemic heart disease, physical inactivity was associated with inadequate management of cardiovascular risk factors, including diabetes, obesity, an unhealthy diet, high LDL-C levels, smoking, and an unhealthy lifestyle overall. Clinical predictors included a history of myocardial infarction or heart failure, a higher GRACE score, and the use of beta-blockers. Psychosocial factors such as low educational attainment, poor knowledge of the disease, low perception of risk, depression, and being female were significant predictors of low activity levels. The association of age was mixed: it was a risk factor in some studies but protective in others. In hypertension, lower physical activity was associated with younger age, female sex, and low self-efficacy.

Reduced participation in cardiac rehabilitation programs was associated with high body mass index, CKD, hypertension, diabetes, older age, and the presence of multiple comorbidities in patients with ischemic heart disease. These factors were more prevalent among females. Additionally, females were less likely to be referred to these programs. Insurance-related barriers were also identified as predictors of non-participation.

Common predictors of non-adherence across different types of secondary prevention included depressive symptoms, anxiety, caregiving burden (particularly among females), CKD, and obesity. Young females were particularly noted for poor control of cardiovascular risk factors. Regarding lifestyle habits, alcohol consumption was identified as a behavior predominantly exhibited by males, who consumed nearly ten times more than females. Smoking was also a key factor: males smoked for longer, but females were more exposed to passive smoking. Overall, both biological and psychosocial factors, which often interact with sex, play a critical role in shaping adherence to secondary prevention measures.

## 4. Discussion

A total of 13 studies from 10 countries involving over 1.1 million individuals were included in the analysis. Females represented only 28% of the sample. Across ischemic heart disease, hypertension, and peripheral arterial disease, females showed consistently lower adherence to pharmacological treatments, physical activity, cardiac rehabilitation, and dietary recommendations. Poor adherence was predicted by younger age, low disease awareness, depression, anxiety, low self-efficacy, and female sex. Females also had lower referral rates to cardiac rehabilitation and poorer adherence to follow-up appointments. Despite having higher smoking cessation rates, females were more affected by passive smoking and caregiving burdens.

This review confirms that sex significantly influences therapeutic adherence in CVD. Across all three conditions reviewed, females exhibited lower adherence rates to both pharmacological and non-pharmacological interventions. The factors contributing to these disparities are multidimensional, encompassing biological, psychological, and social domains.

Although advanced age is generally associated with poorer adherence, some studies have suggested a protective effect in older females, possibly due to increased health awareness and regular use of healthcare services [39,48]. This paradox highlights the need to consider the intersection of age, sex, and social roles as females often face competing demands that influence health behaviors [46]. The burden of comorbidities such as CKD and T2DM is higher in females and contributes to reduced adherence to secondary prevention measures [49,50]. Furthermore, depression, which is up to three times more prevalent in females, was a consistent barrier across studies and was strongly associated with non-adherence [51].

Social expectations and caregiving responsibilities significantly hinder the ability of females to adhere to a prescribed treatment plan. Females are often the primary caregivers within families, a role that can reduce the time, energy, and resources available for managing their own health [36,46]. These findings are consistent with the World Health Organization’s multidimensional model of adherence, which emphasizes the influence of social and sex-related determinants, including family roles, cultural norms, and support systems, on health behaviors [24].

Although our objective was to include qualitative studies, we did not identify any that met our predefined inclusion criteria. In this regard, recent meta-analyses of qualitative research have not examined sex as a variable influencing therapeutic adherence [52]. Some studies have explored barriers perceived by females with cardiovascular disease that contribute to non-participation and dropout from cardiac rehabilitation programs, such as intrapersonal, interpersonal, logistical, programmatic, and health system-related factors, without assessing sex-based differences [53].

To effectively manage CVD, it is essential that healthcare providers adopt a sex-sensitive approach that acknowledges and addresses the distinct clinical, psychosocial, and socioeconomic factors influencing the health behaviors of females. Research has consistently shown that females with CVD are less likely to be referred to or participate in secondary prevention programs, including cardiac rehabilitation, due to caregiving responsibilities, limited social support, and structural barriers to healthcare access [46,54]. Tailored patient education that considers the specific risk profiles, cultural backgrounds, and communication preferences of females can significantly enhance engagement and adherence [55]. Flexible scheduling and delivery models, such as home-based or virtual rehabilitation programs, are particularly beneficial for females who face time constraints due to caregiving responsibilities [56]. Additionally, integrating psychosocial support and mental health counseling into cardiovascular care is critical, given the higher prevalence of depression and anxiety among females, both of which are well-documented barriers to adherence [57]. Routinely incorporating adherence assessments into clinical encounters can help to ensure that individual barriers are identified early and addressed through personalized care planning. Adopting a sex-sensitive framework promotes equity in cardiovascular outcomes and improves the overall effectiveness of secondary prevention strategies.

It is essential to create safe and inclusive environments where females feel empowered to discuss barriers to adherence, such as mental health challenges, caregiving burdens or socioeconomic constraints, without fear of stigma, if we are to effectively prevent cardiovascular disease. Such spaces promote trust, improve communication between patients and providers, and facilitate shared decision-making [58]. To achieve this, collaboration between cardiologists, nurses, psychologists, and social workers is crucial. This team-based approach ensures care is comprehensive, addressing not only clinical indicators, but also psychological well-being and social determinants of health [54,59]. Integrating behavioral health services and social support assessments into routine cardiovascular care has shown promise in enhancing adherence and outcomes among females, particularly those facing multiple vulnerabilities.

Adherence to secondary prevention measures is significantly influenced by cultural and systemic factors. Countries with universal healthcare systems, such as Spain and those in Scandinavia, tend to report higher adherence rates, likely due to reduced financial barriers, more equitable access to care and comprehensive public health strategies [60]. In contrast, studies from the United States highlight the negative effects of financial constraints, particularly among low-income females, who often encounter issues related to insurance coverage, out-of-pocket expenses, and access to supportive services [58,61]. These disparities emphasize the importance of considering national health policies and socioeconomic structures when developing interventions to improve adherence [62].

### 4.1. Limitations

The limitations of this study are primarily determined by the inclusion and exclusion criteria, as well as the search strategy employed. A potential limitation lies in the search strategy, which may have missed relevant qualitative studies addressing sex differences in adherence due to variability in indexing and terminology. This could have contributed to the absence of such studies in our review. Future research should consider incorporating grey literature and expanding the range of databases used in order to capture a broader spectrum of studies, particularly those addressing other conditions, such as heart failure.

A key limitation of this review is its reliance on observational studies, which are inherently limited in establishing causality due to potential confounding, selection bias, and lack of randomization. Therefore the observed associations may not reflect true causal relationships.

A notable limitation of this review is the underrepresentation of females in the included studies, with an average of 28.4% of participants being female. This may constrain the interpretation of sex-related differences in adherence. Future research should aim to recruit more balanced samples to better capture the perspectives and experiences of females.

The lack of standardized definitions and thresholds for adherence across studies, whether related to medication, physical activity, or dietary practices, hinders direct comparison and highlights the need for a consensus on adherence criteria in future research.

As this study is a scoping review, it does not include statistical comparisons of adherence rates between men and women. Further research is required, particularly in the form of systematic reviews and meta-analyses that use standardized adherence measures, in order to quantify sex-based differences in adherence and evaluate their clinical significance.

### 4.2. Implications for Research

The underrepresentation of females in cardiovascular research is a significant obstacle to the development of equitable, evidence-based interventions. Despite increasing recognition of sex-related disparities in CVD outcomes and adherence patterns, females continue to be under-represented in clinical trials and observational studies. Future research must prioritize the inclusion of adequate female cohorts and ensure the systematic collection and reporting of sex-disaggregated data. As well as considering traditional risk factors, investigators should examine contributors to cardiovascular risk that are specific to females, such as gestational hypertension, pre-eclampsia, polycystic ovary syndrome, and the hormonal changes associated with menopause, as these may affect disease progression and treatment adherence in unique ways. Furthermore, interventional trials should evaluate the effectiveness of strategies tailored to enhance adherence for females, such as community-based support models, digital health interventions adapted to caregiving responsibilities, and flexible cardiac rehabilitation formats. Qualitative research is also needed to explore the lived experiences of females with CVD, particularly among marginalized populations, to better understand the complex interplay of biological, psychological, social, and structural determinants of non-adherence. Overall, a sex-informed research agenda is essential to close the evidence gap and improve outcomes for females with CVD.

### 4.3. Conclusions

Sex is a fundamental factor in determining therapeutic adherence to treatment for cardiovascular diseases. Females face unique biological, psychological, and social barriers that can hinder their ability to adhere to prescribed treatments. These disparities require targeted interventions, policy reforms, and ongoing research to ensure equitable cardiovascular care.

Understanding the relationship between sex roles and access to healthcare is essential for improving outcomes for females with CVD. Healthcare systems must prioritize inclusive strategies that recognize and eliminate sex-based barriers to adherence.

## Figures and Tables

**Table 1:** Search strategy.

Database	Search with MeSH and Free Terms	Results
PUBMED	(“Sex Factors” [Mesh]) AND (“Patient Compliance” [Mesh] OR “Medication Adherence” [Mesh] OR “Treatment Adherence and Compliance” [Mesh]) AND (“Cardiovascular Diseases” [Mesh] OR “Arrhythmias, Cardiac” [Mesh] OR “Heart Valve Diseases” [Mesh] OR “Acute Coronary Syndrome” [Mesh] OR “Hypertension, Malignant” [Mesh] OR “Hypertension, Pulmonary” [Mesh] OR “Cardiomyopathies” [Mesh] OR “Heart Failure” [Mesh] OR “Pulmonary Embolism” [Mesh] OR “Pericarditis” [Mesh] OR “Endocarditis” [Mesh] OR “Aortic Diseases” [Mesh] OR “Peripheral Arterial Disease” [Mesh])	63
WEB OF SCIENCE	TS=(“Health Status Disparities” OR “Sex Factors”) AND TS=(“Patient Compliance” OR “Treatment Adherence” OR “Medication Adherence” OR “Compliance” OR “Medication Continuation” OR “Adherence” OR “Therapeutic Compliance”) AND TS=(“Secondary Prevention” OR “Therapeutics”) AND ALL=(“Arrhythmias” OR “Cardiovascular diseases” OR “Heart Valve Diseases” OR “Acute Coronary Syndrome” OR “Chronic Coronary Syndrome” OR “Hypertension” OR “Pulmonary Hypertension” OR “Cardiomyopathies” OR “Heart Failure” OR “Pulmonary Embolism” OR “Pericardial Diseases” OR “Endocarditis” OR “Aortic Diseases” OR “Peripheral Arterial Disease”)	41
SCOPUS	TITLE-ABS-KEY (“Sex Factors”) AND TITLE-ABS-KEY (“Patient Compliance” OR “Treatment Adherence” OR “Medication Adherence” OR “Compliance” OR “Medication Continuation” OR “Adherence” OR “Therapeutic Compliance”) AND ALL (“Therapeutics” OR “Secondary Prevention”) AND ALL (“Arrhythmias” OR “Cardiovascular diseases” OR “Heart Valve Diseases” OR “Acute Coronary Syndrome” OR “Chronic Coronary Syndrome” OR “Hypertension” OR “Pulmonary Hypertension” OR “Cardiomyopathies” OR “Heart Failure” OR “Pulmonary Embolism” OR “Pericardial Diseases” OR “Endocarditis” OR “Aortic Diseases” OR “Peripheral Arterial Disease”)	109

**Table 2:** Summary of adherence prevalence and predictors in males and females.

Authors	Illness	Sample		Results/Sex	Predictors
		N			
Andualem et al. [35]	Hypertension	366	M = 122	Medication adherence: 50.8%	Sex, unemployed occupational status, insufficient knowledge about the disease, poor self-efficacy
			F = 244	Medication adherence: 46.7%	
Moreno et al. [36]	Ischemic heart disease	503	M = 252	M and F at 12 months: - Medication adherence: 32.3% - Adherence to the Mediterranean diet: 56.8% - Physical activity adherence: 71.4% - Cardiac rehabilitation adherence: 33.7% - Global adherence: 15.4%	Overall: HF, peripheral artery disease, stroke, CKD, previous AMI, AF, DM, hypertension, hyperlipidemia, depression, active smoking, burden of family care.
			F = 251		
Consolazio et al. [37]	Hypertension	232,507	M = 128,808	Medication adherence: 82.29%	M: Older age
			F = 103,699	Medication adherence: 80%	F: Younger age
Goodwin et al. [38]	Ischemic heart disease	151	M = 105	Adherence to physical exercise: 68.6%	Overall, the predictors of non-exercise are: older age, female sex, Hispanic ethnicity, worse perceived physical health, higher burden of comorbidities, higher CVR, and history of HF
			F = 46	Adherence to physical exercise: 41.3%	
Hyun et al. [39]	Ischemic heart disease	8761	M = 6244	- Adherence to 75% of the indicated medications: 74% - Current smokers: 14% - Adherence to cardiac rehabilitation: 45%	Overall: Previous AMI, HF, stroke, peripheral artery disease, dyslipidemia, DM, hypertension, active smoking, and CKD
			F = 2517	- Adherence to 75% of the indicated medications: 66% - Current smokers: 13% - Adherence to cardiac rehabilitation: 35%	
Peersen et al. [40]	Ischemic heart disease	1101	M = 872	Adherence to physical activity: 42.7%	Overall: Smoking, low consumption of fruits and vegetables, obesity, depression, and low scores in the physical component of quality of life. Non-modifiable factors: Female sex, low educational level, AMI as an index event, and ≥1 previous coronary event.
			F = 229	Adherence to physical activity: 31.5%	
Haung et al. [41]	Hypertension	410	M = 96	- Adherence to medication: 25% - Adherence to physical activity: 40.7% - Adherence to weight control strategies: 12.5% - Adherence to smoking cessation strategies: 42.7%	In general, in both sexes: being younger, living alone, less education, having a low family income, and residing in a rural area
			F = 314	- Adherence to medication: 23.9% - Adherence to physical activity: 20.1% - Adherence to weight control strategies: 8.6% - Adherence to smoking cessation strategies: 52.5%	
Wawruch et al. [42]	Peripheral artery disease	8330	M = 3433	Medication adherence: 70.9%	In general, for both sexes: Patients starting atorvastatin or rosuvastatin therapy, being a new statin user, having hypercholesterolemia, depression, anxiety disorders, being female, receiving an increased co-pay (covered medications), and having a mild–moderate intensity of treatment
			F = 4897	Medication adherence: 59.6%	
Setny et al. [43]	Ischemic heart disease	1236	M = 882	- Adherence to medical check-ups: 72% - Percentage of goals achieved: LDL control (25%) Smoking cessation: 44% Adherence to physical activity: 21% Prevalence of central obesity: 16% BP control: 57% HbA1c < 7%: 63% BMI within the normal range: 23%	M: Active smoking and overweight.
			F = 354	- Adherence to medical check-ups: 28% - Percentage of goals achieved: LDL control: 20% Smoking cessation: 46% Adherence to physical activity: 14% Prevalence of central obesity: 5% BP control: 57% HbA1c < 7%: 61%	F: Higher burden of risk factors (59% had 3 or more CVRF), central obesity, passive smoking in young females; almost twice as many of them had a reduced glomerular filtration rate and high anxiety.
Mahtta et al. [44]	Ischemic heart disease	484,134	M = 471,319	- Medication adherence with peripheral arterial disease: 45.46%	M: Higher prevalence of hypertension, DM, ischemic heart disease, AMI
			F = 12,815	- Medication adherence with peripheral arterial disease: 34.56%	F: Higher levels of LDL and HDL, as well as higher burden of disease (which means a higher cost of medical care compared to males)
Hojskov et al. [45]	Ischemic heart disease	152	M = 132	- Adherence to hospital physical activity: 80% - Adherence to post-discharge physical activity: 30%	Of both sexes: obesity, diabetes and antidiabetic therapy, use of beta-blockers, lower educational level
			F = 20	- Adherence to hospital physical activity: 69% - Adherence to post-discharge physical activity: 47%	
Ritchey et al. [46]	Ischemic heart disease	366,103	M = 207,911	- Participation in cardiac rehabilitation: 28.6% - Completion of ≥36 sessions: 28.3%	Adults 65–74 years old, dual eligible (receive financial aid)
			F = 158,192	- Participation in cardiac rehabilitation: 18.9% - Completion of ≥36 sessions: 26.1%	
Rea et al. [47]	Hypertension	60,529	M = 30,860	- Medication adherence: 53% - Discontinuation of treatment during follow-up: 33.8%	M: DM and respiratory diseases
			F = 29,666	- Medication adherence: 42% - Discontinuation of treatment during follow-up: 44.1%	F: Antidepressant use, cancer

Note: AF: atrial fibrillation; AMI: acute myocardial infarction; BP: blood pressure; CKD: chronic kidney disease; CVRF: cardiovascular risk factors; DM: diabetes mellitus; F: female; HDL: high density lipoprotein; HF: heart failure; LDL: low density lipoprotein; M: male.

## Data Availability

The datasets generated and/or analyzed during the current study are not publicly available, but are available from the corresponding author (G.M.) upon reasonable request.

## Supplementary Materials

The following supporting information can be downloaded at: https://www.mdpi.com/article/10.3390/jcm14124253/s1, Table S1: Assessment of the quality level of the articles included using CASPe.

## References

[1] Cardiovascular Diseases (CVDs). https://www.who.int/news-room/fact-sheets/detail/cardiovascular-diseases-(cvds).

[2] Statistics on Deaths by Cause of Death. Year 2023. https://www.ine.es/dyngs/Prensa/pEDCM2023.htm.

[3] Romeo B., Bergami M., Cenko E., Manfrini O., Bugiardini R. (2024). Sex Disparities in Ischemic Heart Disease Mortality in Europe, 2005–2019: Data from GBD and Eurostat. JACC Adv..

[4] Humphries K., Izadnegahdar M., Sedlak T., Saw J., Johnston N., Schenck-Gustafsson K., Shah R., Regitz-Zagrosek V., Grewal J., Vaccarino V. (2017). Sex differences in cardiovascular disease—Impact on care and outcomes. Front. Neuroendocr..

[5] Ng M., Fleming T., Robinson M., Thomson B., Graetz N., Margono C., Mullany E.C., Biryukov S., Abbafati C., Abera S.F. (2014). Global, regional, and national prevalence of overweight and obesity in children and adults during 1980–2013: A systematic analysis for the Global Burden of Disease Study 2013. Lancet.

[6] Maas A.H.E.M., Appelman Y.E.A. (2010). Gender differences in coronary heart disease. Neth. Heart J..

[7] Reckelhoff J.F. (2001). Gender differences in the regulation of blood pressure. Hypertension.

[8] Stramba-Badiale M., Fox K.M., Priori S.G., Collins P., Daly C., Graham I., Jonsson B., Schenck-Gustafsson K., Tendera M. (2006). Cardiovascular diseases in women: A statement from the policy conference of the European Society of Cardiology. Eur. Heart J..

[9] Peters S.A.E., Huxley R.R., Woodward M. (2014). Diabetes as risk factor for incident coronary heart disease in women compared with men: A systematic review and meta-analysis of 64 cohorts including 858,507 individuals and 28,203 coronary events. Diabetologia.

[10] Huxley R., Barzi F., Woodward M. (2006). Excess risk of fatal coronary heart disease associated with diabetes in men and women: Meta-analysis of 37 prospective cohort studies. BMJ.

[11] Remsberg K.E., Demerath E.W., Schubert C.M., Chumlea W.C., Sun S.S., Siervogel R.M. (2005). Early menarche and the development of cardiovascular disease risk factors in adolescent girls: The Fels Longitudinal Study. J. Clin. Endocrinol. Metab..

[12] Wilson P.W.F. (2006). Smoking, smoking cessation, and risk of cardiovascular disease. Curr. Treat Options Cardiovasc. Med..

[13] McArthur D., Dumas A., Woodend K., Beach S., Stacey D. (2014). Factors influencing adherence to regular exercise in middle-aged women: A qualitative study to inform clinical practice. BMC Womens Health.

[14] Britton L.E., Kaur G., Zork N., Marshall C.J., George M. (2023). ‘We tend to prioritise others and forget ourselves’: How women’s caregiving responsibilities can facilitate or impede diabetes self-management. Diabet. Med..

[15] Cederlöf E.T., Lundgren M., Lindahl B., Christersson C. (2022). Pregnancy Complications and Risk of Cardiovascular Disease Later in Life: A Nationwide Cohort Study. J. Am. Heart Assoc..

[16] Gunderson E.P., Sun B., Catov J.M., Carnethon M., Lewis C.E., Allen N.B., Sidney S., Wellons M., Rana J.S., Hou L. (2021). Gestational Diabetes History and Glucose Tolerance After Pregnancy Associated With Coronary Artery Calcium in Women During Midlife The CARDIA Study. Circulation.

[17] Mendelsohn M.E., Karas R.H. (1999). The Protective Effects of Estrogen on the Cardiovascular System. N. Engl. J. Med..

[18] Manson J.A.E., Chlebowski R.T., Stefanick M.L., Aragaki A.K., Rossouw J.E., Prentice R.L., Anderson G., Howard B.V., Thomson C.A., LaCroix A.Z. (2013). Menopausal hormone therapy and health outcomes during the intervention and extended poststopping phases of the women’s health initiative randomized trials. JAMA.

[19] Colella T.J., Gravely S., Marzolini S., Grace S.L., Francis J.A., Oh P., Scott L.B. (2015). Sex bias in referral of women to outpatient cardiac rehabilitation? A meta-analysis. Eur. J. Prev. Cardiol..

[20] Arber S., Ginn J. (1993). Gender and inequalities in health in later life. Soc. Sci. Med..

[21] Healy B. (1991). The Yentl syndrome. N. Engl. J. Med..

[22] Smith J.R., Thomas R.J., Bonikowske A.R., Hammer S.M., Olson T.P. (2022). Sex Differences in Cardiac Rehabilitation Outcomes. Circ. Res..

[23] Aljofan M., Oshibayeva A., Moldaliyev I., Saruarov Y., Maulenkul T., Gaipov A. (2023). The rate of medication nonadherence and influencing factors: A systematic Review. Electron. J. Gen. Med..

[24] World Health Organization (2003). Adherence to Long-Term Therapies: Evidence for Action. https://iris.who.int/handle/10665/42682.

[25] Venditti V., Bleve E., Morano S., Filardi T. (2023). Gender-Related Factors in Medication Adherence for Metabolic and Cardiovascular Health. Metabolites.

[26] Dean Y.E., Motawea K.R., Shebl M.A., Elawady S.S., Nuhu K., Abuzuaiter B., Awayda K., Fouad A.M., Tanas Y., Batista R. (2024). Adherence to antihypertensives in the United States: A comparative meta-analysis of 23 million patients. J. Clin. Hypertens..

[27] Kokenge M.C., Ruppar T.M. (2024). Factors Influencing Antihypertensive Medication Adherence among Historically Underrepresented Adults: A Meta-analysis. J. Cardiovasc. Nurs..

[28] Abegaz T.M., Shehab A., Gebreyohannes E.A., Bhagavathula A.S., Elnour A.A. (2017). Nonadherence to antihypertensive drugs a systematic review and meta-analysis. Medicine.

[29] Olmastroni E., Boccalari M.T., Tragni E., Rea F., Merlino L., Corrao G., Catapano A.L., Casula M. (2020). Sex-differences in factors and outcomes associated with adherence to statin therapy in primary care: Need for customisation strategies. Pharmacol. Res..

[30] Lewey J., Shrank W.H., Bowry A.D.K., Kilabuk E., Brennan T.A., Choudhry N.K. (2013). Gender and racial disparities in adherence to statin therapy: A meta-analysis. Am. Heart J..

[31] Oosenbrug E., Marinho R.P., Zhang J., Marzolini S., Colella T.J.F., Pakosh M., Grace S.L. (2016). Sex Differences in Cardiac Rehabilitation Adherence: A Meta-analysis. Can. J. Cardiol..

[32] Resurrección D.M., Moreno-Peral P., Gómez-Herranz M., Rubio-Valera M., Pastor L., de Almeida J.M.C., Motrico E. (2019). Factors associated with non-participation in and dropout from cardiac rehabilitation programmes: A systematic review of prospective cohort studies. Eur. J. Cardiovasc. Nurs..

[33] Moher D., Liberati A., Tetzlaff J., Altman D.G., Antes G., Atkins D., Prisma Group (2009). Preferred reporting items for systematic reviews and meta-analyses: The PRISMA statement. PLoS Med..

[34] Redcaspe—Critical Appraisal Skills Programme Español. https://redcaspe.org/.

[35] Andualem A., Liknaw T., Edmealem A., Gedefaw M. (2021). Adherence to antihypertensive medications among adult hypertensive patients attending chronic follow-up units of Dessie Referral Hospital, Northeastern Ethiopia: A cross-sectional study. Medicine.

[36] Moreno G., Vicent L., Rosillo N., Delgado J., Cerro EPDel Bueno H. (2024). Do sex and gender aspects influence non-adherence to secondary prevention measures after myocardial infarction?. Am. J. Prev. Cardiol..

[37] Consolazio D., Gattoni M.E., Russo A.G. (2022). Exploring gender differences in medication consumption and mortality in a cohort of hypertensive patients in Northern Italy. BMC Public Health.

[38] Goodwin A.M., Duran A.T., Kronish I.M., Moise N., Sanchez G.J., Garber C.E., Schwartz J.E., Diaz K.M. (2019). Factors associated with objectively measured exercise participation after hospitalization for acute coronary syndrome. Int. J. Cardiol..

[39] Hyun K., Negrone A., Redfern J., Atkins E., Chow C., Kilian J., Rajaratnam R., Brieger D. (2021). Gender Difference in Secondary Prevention of Cardiovascular Disease and Outcomes Following the Survival of Acute Coronary Syndrome. Heart Lung Circ..

[40] Peersen K., Otterstad J.E., Sverre E., Perk J., Gullestad L., Moum T., Dammen T., Munkhaugen J. (2020). Medical and Psychosocial Factors Associated with Low Physical Activity and Increasing Exercise Level after a Coronary Event. J. Cardiopulm. Rehabil. Prev..

[41] Haung Z., Hong S.A., Tejativaddhana P., Puckpinyo A., Myint M.N.H.A. (2020). Multiple self-care behaviors and associated factors in community-dwelling patients with hypertension in Myanmar. Nagoya J. Med. Sci..

[42] Wawruch M., Wimmer G., Murin J., Paduchova M., Tesar T., Hlinkova L., Slavkovsky P., Fabryova L., Aarnio E. (2019). Patient-Associated Characteristics Influencing the Risk for Non-Persistence with Statins in Older Patients with Peripheral Arterial Disease. Drugs Aging.

[43] Setny M., Jankowski P., Kaminski K., Gasior Z., Haberka M., Czarnecka D., Pająk A., Kozieł P., Szóstak-Janiak K., Sawicka E. (2021). Secondary prevention of coronary heart disease in Poland: Does sex matter? Results from the POLASPIRE survey. Pol. Arch. Intern. Med..

[44] Mahtta D., Ahmed S.T., Ramsey D.J., Akeroyd J.M., Lee M.T., Rodriguez F., Michos E.D., Itchhaporia D., Nasir K., Alam M. (2020). Statin Prescription Rates, Adherence, and Associated Clinical Outcomes Among Women with PAD and ICVD. Cardiovasc. Drugs Ther..

[45] Højskov I.E., Thygesen L.C., Moons P., Egerod I., Olsen P.S., Berg S.K. (2020). The challenge of non-adherence to early rehabilitation after coronary artery bypass surgery: Secondary results from the SheppHeartCABG trial. Eur. J. Cardiovasc. Nurs..

[46] Ritchey M.D., Maresh S., McNeely J., Shaffer T., Jackson S.L., Keteyian S.J., Brawner C.A., Whooley M.A., Chang T., Stolp H. (2020). Tracking cardiac rehabilitation participation and completion among Medicare beneficiaries to inform the efforts of a national initiative. Circ. Cardiovasc. Qual. Outcomes.

[47] Rea F., Mella M., Monzio Compagnoni M., Cantarutti A., Merlino L., Mancia G., Corrao G. (2020). Women discontinue antihypertensive drug therapy more than men. Evidence from an Italian population-based study. J. Hypertens..

[48] Perera S., Aslam A., Stehli J., Kaye D., Layland J., Nicholls S.J., Cameron J., Zaman S. (2021). Gender Differences in Healthy Lifestyle Adherence Following Percutaneous Coronary Intervention for Coronary Artery Disease. Heart Lung Circ..

[49] Khan N.N.S., Kelly-Blake K., Luo Z., Olomu A. (2019). Sex Differences in Statin Prescribing in Diabetic and Heart Disease Patients in FQHCs: A Comparison of the ATPIII and 2013 ACC/AHA Cholesterol Guidelines. Health Serv. Res. Manag. Epidemiol..

[50] Mehta L.S., Velarde G.P., Lewey J., Sharma G., Bond R.M., Navas-Acien A., Fretts A.M., Magwood G.S., Yang E., Blumenthal R.S. (2023). Cardiovascular Disease Risk Factors in Women: The Impact of Race and Ethnicity: A Scientific Statement from the American Heart Association. Circulation.

[51] Bautista L.E., Vera-Cala L.M., Colombo C., Smith P. (2012). Symptoms of depression and anxiety and adherence to antihypertensive medication. Am. J. Hypertens..

[52] Marshall I.J., Wolfe C.D.A., McKevitt C. (2012). Lay perspectives on hypertension and drug adherence: Systematic review of qualitative research. BMJ.

[53] Resurrección D.M., Motrico E., Rigabert A., Rubio-Valera M., Conejo-Cerón S., Pastor L., Moreno-Peral P. (2017). Barriers for Nonparticipation and Dropout of Women in Cardiac Rehabilitation Programs: A Systematic Review. J. Women’s Health.

[54] Mosca L., Barrett-Connor E., Kass Wenger N. (2011). Sex/gender differences in cardiovascular disease prevention: What a difference a decade makes. Circulation.

[55] Mosca L., Mochari-Greenberger H., Dolor R.J., Newby L.K., Robb K.J. (2010). Twelve-year follow-up of American women’s awareness of cardiovascular disease risk and barriers to heart health. Circ. Cardiovasc. Qual. Outcomes.

[56] Clark A.M., King-Shier K.M., Duncan A., Spaling M., Stone J.A., Jaglal S., Angus J. (2013). Factors influencing referral to cardiac rehabilitation and secondary prevention programs: A systematic review. Eur. J. Prev. Cardiol..

[57] Smolderen K.G., Strait K.M., Dreyer R.P., D’Onofrio G., Zhou S., Lichtman J.H., Geda M., Bueno H., Beltrame J., Safdar B. (2015). Depressive symptoms in younger women and men with acute myocardial infarction: Insights from the VIRGO study. J. Am. Heart Assoc..

[58] Ferreruela I.L., Azuara B.O., Fumanal S.M., Hernández M.J.R., Aguilar-Palacio I. (2024). Gender inequalities in secondary prevention of cardiovascular disease: A scoping review. Int. J. Equity Health.

[59] Vaccarino V., Sullivan S., Hammadah M., Wilmot K., Al Mheid I., Ramadan R., Elon L., Pimple P.M., Garcia E.V., Nye J. (2018). Mental stress-induced-myocardial ischemia in young patients with recent myocardial infarction: Sex differences and mechanisms. Circulation.

[60] Baigent C., Keech A., Kearney P.M., Blackwell L., Buck G., Pollicino C., Kirby A., Sourjina T., Peto R., Collins R. (2005). Efficacy and safety of cholesterol-lowering treatment: Prospective meta-analysis of data from 90,056 participants in 14 randomised trials of statins. Lancet.

[61] Havranek E.P., Mujahid M.S., Barr D.A., Blair I.V., Cohen M.S., Cruz-Flores S., Davey-Smith G., Dennison-Himmelfarb C.R., Lauer M.S., Lockwood D.W. (2015). Social determinants of risk and outcomes for cardiovascular disease: A scientific statement from the American Heart Association. Circulation.

[62] Lee H., Park J.H., Floyd J.S., Park S., Kim H.C. (2019). Combined Effect of Income and Medication Adherence on Mortality in Newly Treated Hypertension: Nationwide Study of 16 Million Person-Years. J. Am. Heart Assoc..

