# Supplementary material for: Influence of Sex on Therapeutic Adherence in Cardiovascular Diseases: A Scoping Review
Source: J Clin Med. 2025 Jun 15;14(12):4253. doi: 10.3390/jcm14124253 (PMC12194201; doi:10.3390/jcm14124253)
Supplement: Supplementary file 1 [file jcm-14-04253-s001.zip › jcm-3674192-supplementary.pdf]

Supplementary

Supplementary Table S1. Assessment of the quality level of the articles included using CASPe.

	Quality assesment score using CASPe
Sex-Specific Considerations in the Presentation, Diagnosis and Management of Ischemic Heart Disease; *S. Solola-Nussbaum, S. Henry, C.M. Yong, S. L. Daugherty, R. Mehran, A. Poppas*	6
Gender inequalities in secondary prevention of cardiovascular disease: a scoping review; *I. López Ferreruela, B. Obón Azuara, S. Malo Fumanal, M.J. Rabanaque Hernández, I. Aguilar-Palacio*	8
Nature, availability, and utilization of females-focused cardiac rehabilitation: a systematic review; *T. Mamataz, G. L. M. Ghisi, M. Pakosh, S. L. Grace*	8
Sex and Gender Aspects in Vascular Ageing – Focus on Epidemiology, Pathophysiology, and Outcomes; *U. Seeland, J. Nemcsik, M.T. Lønnebakken, K. Kublickiene, H. Schluchter, C. Park, G. Pucci, I. Mozos, R.M. Bruno*	7
Sex-Related Disparities in Cardiovascular Health Care Among Patients With Premature Atherosclerotic Cardiovascular Disease; *M.T. Lee, D. Mahtta, D.J. Ramsey, J. Liu, A. Misra*	5
Tracking Cardiac Rehabilitation Participation and Completion Among Medicare Beneficiaries to Inform the Efforts of a National Initiative: *M.D. Ritchey, S. Maresh, J. McNeely, T, Shaffer, S.L. Jackson, S. J. Keteyian, C.A. Brawner, M.A. Whooley, T. Chang, H. Stolp, L. Schieb, J. Wright*	10
The challenge of non-adherence to early rehabilitation after coronary artery bypass surgery: Secondary results from the SheppHeartCABG trial; *I.E Højskov, L. C. Thygesen, P. Moons, I. Egerod, P. Olsen, S. K. Berg*	8
Females discontinue antihypertensive drug therapymore thanmen. Evidence froman Italian population-based study; *F. Rea, M. Mellab, M. M. Compagnoni, A. Cantaruttia, L. Merlinoc, G. Mancia, G. Corrao*	10
Exploring gender differences in medication consumption and mortality in a cohort of hypertensive patients in Northern Italy: *D. Consolazio, M.E. Gattoni, A. G. Russo*	10
Statin Prescription Rates, Adherence, and Associated Clinical Outcomes Among Females with PAD and ICVD; *D. Mahtta, S.T. Ahmed, D.J. Ramsey, J.M. Akeroyd, M.T. Lee, F. Rodriguez, E.D. Michos, D. Itchhaporia, K. Nasir, M. Alam, H. Jneid, C.M. Ballantyne, L.A. Petersen, S.S. Virani*	9
Do sex and gender aspects influence non-adherence to secondary prevention measures after myocardial infarction?; *G. Moreno, L. Vicent, N. Rosillo, J. Delgado, E. Pacheco Del Cerro, H. Bueno*	9
Secondary prevention of coronary heart disease in Poland: does sex matter? Results from the POLASPIRE survey; *M. Setny, P. Jankowski, K. Kaminoski, Z. Gasior, M. Haberka, D. Czarnecka, A. Pająk, P. Kozieł, K. Szóstak Janiak, E. Sawicka, Z. Stachurska, D.A. Kosior*	10
Multiple self-care behaviors and associated factors in community-dwelling patients with hypertension in Myanmar: *Z. Haung, S.A. Hong, P. Tejativaddhana, A. Puckpinyo, M.N.H.A. Myint*	8
Patient-Associated Characteristics Influencing the Risk for Non-Persistence with Statins in Older Patients with Peripheral Arterial Disease; *M. Wawruch, G. Wimmer, J. Murin, M. Paduchova, T. Tesar, L. Hlinkova, P. Slavkovsky, L. Fabryova, E. Aarnio*	9
Medication Adherence among Hypertensive Patients Attending a Tertiary Care Hospital in Nepal; *T. Roka, M. Ghimire.*	6

Depression and medication-adherence in patients with hypertension attending a tertiary health facility in South-West Nigeria; *H Iyabo Okunrinboye. A. Ndubusi Otakpor, O. Stephen Ilesanmi*	6
Medical and Psychosocial Factors Associated With Low Physical Activity and Increasing Exercise Level After a Coronary Event; *K. Peersen, J.E. Otterstad, E. Sverre, J. Perk, L. Gullestad, T. Moum , T. Dammen, J. Munkhau*	9
Achievement of European Society of Cardiology/European Atherosclerosis Society lipid targets in very high-risk patients: Influence of depression and sex; *E.A. Ellins, D.E. Harris, A. Lacey, A. Akbari, F. Torabi, D. Smith, G. Jenkins, D. Obaid, A. Chase, A, John, M. B. Gravenor, J. P. Halcox*	7
Adherence to antihypertensive medications among adult hypertensive patients attending chronic follow-up units of Dessie Referral Hospital, Northeastern Ethiopia. A cross-sectional study; *A. Andualem, T. Liknaw, A. Edmealem, M. Gedefaw,*	8
Gender Difference in Secondary Prevention of Cardiovascular Disease and Outcomes Following the Survival of Acute Coronary Syndrome; *K. Hyun, A. Negrone, J. Redfern, E. Atkins, C. Chow, J. Kilian, R. Rajaratnam, D. Briege*	9
Factors associated with objectively measured exercise participation after hospitalization for acute coronary syndrome; A.M. Goodwin, A.T. Duran, I.M. Kronish, N. Moise, G.J. Sanchez, C.E. Garber, J.E. Schwartz, K.M. Diaz	9
Sex-based disparities with cost-related medication adherence issues in patients with hypertension, ischemic heart disease, and heart failure, *I. Agapito , T. Hoang, M. Sayer , A. Naqvi, P. M. Patel, A.F. Ozaki*	7
The Effect of Contextualized Racial and Gendered Stressors, Social Support, and Depression on Hypertension IIlness Perceptions and Hypertension Medication Adherence in Young African American Females With Hypertension; *T. Spikes , M. Higgins, T. Lewis, S. Dunbar*	7
